# The complete chloroplast genome of *Ligusticum jeholense* (Umbelliferae, *Ligusticum* L.)

**DOI:** 10.1080/23802359.2020.1797560

**Published:** 2020-07-28

**Authors:** Shi-Jie Wang, Ting-Ting Zhang, Liang Xu, Yan-Yun Yang, Ming Xie, Ting-Guo Kang

**Affiliations:** School of Pharmacy, Liaoning University of Traditional Chinese Medicine, Dalian, China

**Keywords:** Chloroplast genome, phylogenetic tree, Umbelliferae, *Ligusticum jeholense*

## Abstract

*Ligusticum jeholense* is an important medicinal plant. Chloroplast genome information is helpful for the development of molecular markers and the study of plant phylogeny. In this study, we report the complete chloroplast genome sequence of *L. jeholense*. The genome sequence is 148,493 bp in size (GenBank accession number MN652885), with 37.25% GC contents. There are 127 genes in the genome, including 83 known protein-coding genes (PCGs), 36 transfer RNAs (tRNAs), and 8 ribosomal RNAs (rRNAs). The maximum-likelihood method are used to construct phylogenetic tree of 32 species. These data will provide certain theoretical basis for plant genetics research.

Umbelliferae is a family under Umbelliflorae. There are more than 200 genera and 2500 species in the world, which are widely distributed in the tropical and temperate climatic zone. Many Umbelliferae plants are used as medicinal plants, essential oil plants, and vegetables. Therefore, they are considered to be one of the most valuable agricultural families among angiosperms. But, at present, there are still some difficulties in the systematic research of Umbelliferae (Degtjareva et al. 2012). *Ligusticum jeholense* is a member of the Umbelliferae plant, which is called Liao GaoBen (LGB) in China. Its rhizome can be used as a traditional Chinese medicine, which has the effects of dispelling wind, dispersing cold, dehumidifying and analgesic (National Pharmacopoeia Commission. 2015). Modern pharmacological studies have shown that the roots and rhizomes of *L. jeholense* can relax blood vessels and have a certain effect on the treatment of hypertension (Kim et al. 2015). In addition, the aerial part of *L. jeholense* also contains some chemical components, which also have certain pharmacological action (Sun et al. 2011). Chloroplast genome sequence can provide important reference value for plant identification and evolution research (Zhang et al. 2019; Xing et al. 2019). Previously, the chloroplast genome information of *L. jeholense* has not been reported.

In order to obtain more molecular level information, the chloroplast genome sequence of *L. jeholense* was sequenced, assembled and annotated for the first time. Fresh leaves of *L. jeholense* were collected from Dalian, China (121°87′73.84″E, 39°06′31.81″N). Professor Xu Liang of Liaoning University of Traditional Chinese Medicine identified the certified specimen (*L. jeholense* number: 10162190502010LY). The plant samples were deposited at the herbarium of Liaoning University of Traditional Chinese Medicine, and the genomic DNA were stored in the Key Laboratory of Traditional Chinese Medicine. The chloroplast genome information of *L. jeholense* is helpful for phylogenetic studies of Umbelliferae.

The chloroplast genome sequence of *L. jeholense* was 148,493 bp in length (GenBank accession number MN652885), and GC content was 37.25%. The genome had a typical quadripartite structure. The length of large single-copy (LSC), small single-copy (SSC), and inverted repeat (IR) regions were 93,932, 17,629, and 36,932 bp, respectively. Totally 127 genes were annotated, including 83 known protein-coding genes, 36 tRNAs, and 8 rRNAs. The total length of protein-coding genes was 70,617 bp, accounting for 47.56% of the total genome length. The total length of tRNA was 2,707 bp, while rRNA was 9,046 bp. Among them, seven genes contained one intron, while three had two introns.

Using the maximum-likelihood method, based on the complete chloroplast genome, a phylogenetic tree of 32 species was constructed, including 30 Umbelliferae plants (including *L. jeholense*, LGB) and two species (*Arabidopsis thaliana* and *Ginkgo biloba*) of the outgroup. The result of the tree was supported by high bootstrap values. The accession numbers of chloroplast genome obtained from NCBI are shown in the tree. The evolutionary relationship between *L. jeholense* and Umbelliferae plants was vividly reflected. Additionally, 2 outgroup species were far from the other species (Figure 1). The construction of phylogenetic tree will provide scientific basis for the research on the evolution of the Umbelliferae.

**Figure 1.**
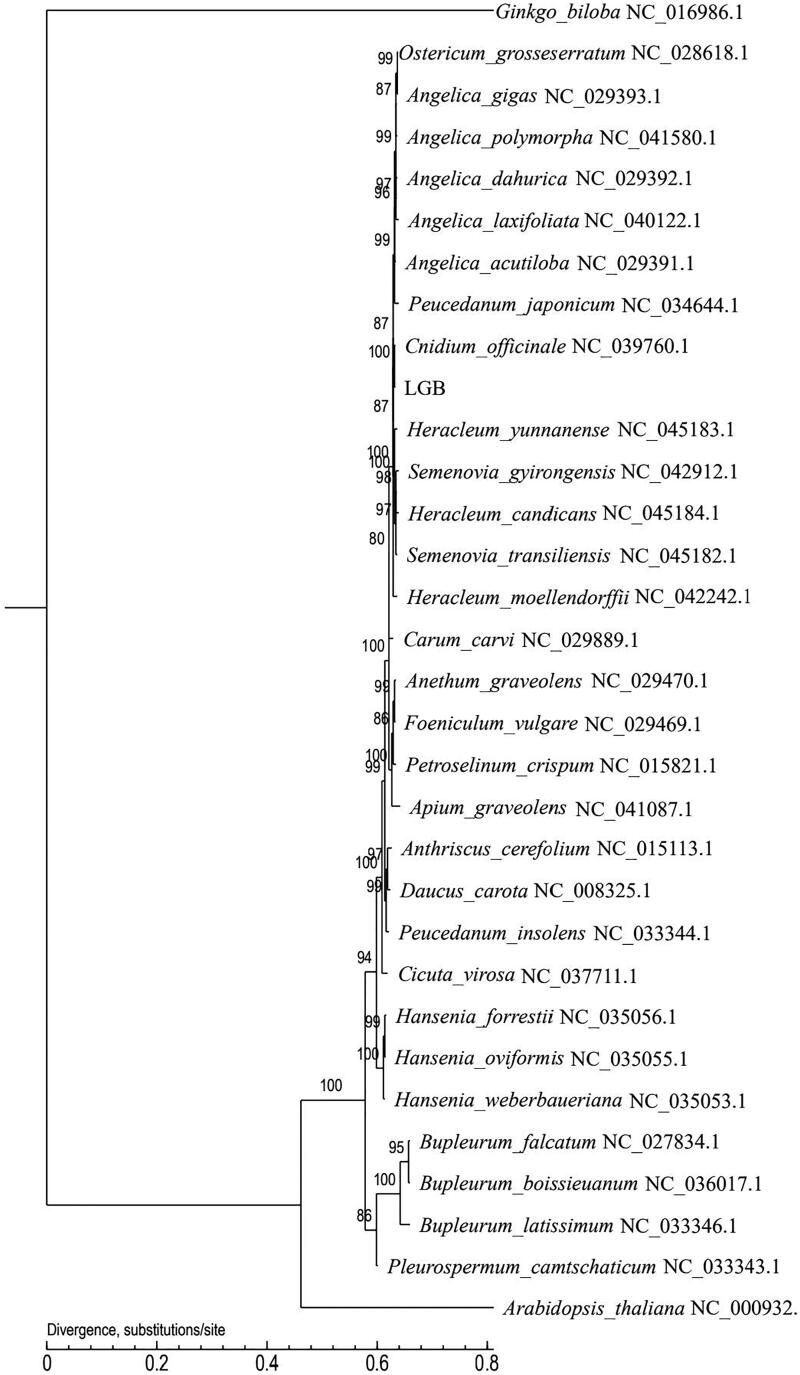
Maximum-likelihood (ML) phylogenetic tree based on the chloroplast sequence of *L. jeholense* (LGB) and 31 other species. Number above each node indicates the ML bootstrap support values.

## Data Availability

The data that support the findings of this study are openly available in NCBI at https://www.ncbi.nlm.nih.gov/search/all/?term=MN652885, reference number MN652885.
